# Effect of different cooking methods on the sensory, nutritional, and flavor profiles of three *Cucurbita moschata* and *Cucurbita maxima* cultivars

**DOI:** 10.1016/j.fochx.2026.103611

**Published:** 2026-01-30

**Authors:** Shudan Xue, Yang Yu, Yingchao Xu, Wenlong Luo, Yingyin Lin, Muhammad Sajjad, Qingmin Jin, Dasen Xie, Yujuan Zhong

**Affiliations:** Guangdong Key Laboratory for New Technology Research of Vegetables, Vegetable Research Institute, Guangdong Academy of Agricultural Sciences, Guangzhou, Guangdong 510640, China

**Keywords:** Pumpkin, Cooking methods, Texture, Organic acids, Carotenoids, Vitamin C, DPPH, Aroma compounds

## Abstract

Pumpkin (*Cucurbita moschata* and *Cucurbita maxima*) is a nutrient-rich vegetable, yet cooking-induced changes in comprehensive quality attributes across cultivars remain poorly understood. Here, the effects of steaming, boiling, and baking on taste components, texture, nutritional quality, and volatile profiles were systematically evaluated in three pumpkin cultivars. Sugar and starch contents were largely unaffected by cooking, whereas textural properties were significantly altered, with baking causing the least structural disruption. Steaming and boiling increased organic acid and vitamin C contents as well as antioxidant activity. Boiling enhanced carotenoid extractability, with *Tian Mi* highest at 3.13 mg/g DW. Cultivar-dependent differences were observed: *Dan Hong* showed higher hardness, chewiness, and gumminess, while *Zhen Mi* exhibited greater total volatile content. Volatile analysis revealed that cooking, particularly boiling, reduced green and earthy odorants while increasing key aroma-active compounds, including furfural and 4-vinylguaiacol. Overall, boiling provided the most favorable balance between nutritional enhancement and aroma improvement.

## Highlights

• Cooking methods differently affect pumpkin texture; baking causes the least change.
• Steaming and boiling increase organic acids, potentially reducing taste quality.
• Boiling enhances carotenoids, reaching 3.13 mg/g DW in *C. moschata* TM.
• Steaming and boiling improve vitamin C content and antioxidant activity.
• Boiling reduces green/earthy notes and increases sweet, caramel-like aroma compounds.

## Graphical abstract

**Figure:**
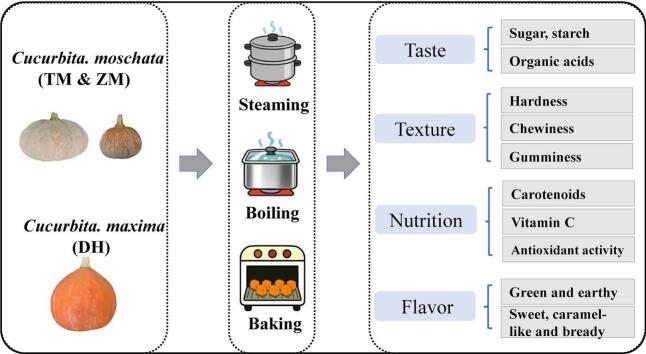

## 1 Introduction

The *Cucurbita* genus (Cucurbitaceae) contains 15–20 taxa, among which three domesticated species, *Cucurbita pepo*, *Cucurbita maxima*, and *Cucurbita moschata*, are widely cultivated around the world. These pumpkins and squashes are not only grown across diverse agro-climatic regions but also hold substantial economic value as staple vegetable crops (Hosen et al., 2021). The value of the global market for these vegetables is estimated to be 9.104 billion USD, and Asia accounts for 50.5% of their total production. China is the largest producer of these vegetables, yielding over 7.3 million tons (FAOSTAT, 2022; https://www.fao.org/faostat/en/#data/QCL). As unique members of the Cucurbitaceae family, pumpkins are recognized for their high starch content, which can reach up to 60% of their dry weight (Yuan et al., 2022). They are also abundant in functional metabolites such as polysaccharides, carotenoids, flavonoids, phenolic compounds, inositols, dietary fiber, vitamins, and minerals, which strengthen immunity and contribute to disease resistance (Piepiórka-Stepuk et al., 2023).

Different species of pumpkin contain a distinct set of compounds, which determine their final texture, taste, and flavor after cooking. A range of cooking and processing methods is employed to facilitate the year-round utilization of pumpkins, including boiling, steaming, baking, microwaving, sous vide, stir-frying, drying, pickling, and even raw consumption (Mashiane et al., 2021). *Cucurbita moschata* and *Cucurbita maxima* are starchy pumpkins commonly consumed as mature fruits, which can be cooked by steaming, boiling, or baking, while *Cucurbita pepo* is generally harvested and eaten as an immature fruit. Considerable researches have investigated how cooking affects the nutritional and sensory properties of plant-based foods, including sweet potato, chestnut, cauliflower, and carrot. The alterations in physical and chemical characteristics caused by different cooking methods differ among crops, exhibiting both common patterns and crop-specific responses, which in turn directly impact their nutritional quality and sensory attributes (Buratti et al., 2020; Gonçalves et al., 2010; Kotíková et al., 2016). Currently, some studies have reported the effects of cooking on pumpkin quality, primarily focusing on color (Piepiórka-Stepuk et al., 2023), the content of bioactive compounds (such as carotenoids, vitamin C, and polyphenols) (Monalisa et al., 2019; Moreira et al., 2020; Piepiórka-Stepuk et al., 2023), antioxidant properties (DPPH radical scavenging activity) (Rinaldi et al., 2021), and texture (Moreno et al., 2023; Rinaldi et al., 2021). However, prior studies have generally relied on a narrow selection of cultivars and single cooking treatments, and have yet to provide a comprehensive, comparative assessment of the impact of diverse cooking methods on pumpkin. More importantly, aroma also plays a crucial role in shaping the overall sensory quality of food, significantly influencing taste perception and the enjoyability of a dish. The volatile compounds released during cooking are detected by the olfactory system, contributing to overall flavor and making food more appetizing. Most studies to date have focused on flavor compounds in raw pumpkin, with limited investigation of their transformations during cooking (Hagos et al., 2022; Leffingwell et al., 2015; Li et al., 2022; Zhou et al., 2017). Since the flavor developed through thermal processing ultimately determines consumer perception, it is essential to systematically examine how pumpkin flavor evolves during cooking. To date, systematic studies comparing the effects of different cooking methods across multiple Cucurbita cultivars on comprehensive quality attributes, including taste, texture, nutrition, and flavor, are still lacking.

Therefore, the objective of this study was to systematically compare the impact of boiling, steaming, and baking on the nutritional quality, sensory acceptance, and volatile flavor profiles of three pumpkin cultivars (*C. moschata* and *C. maxima*). Specifically, we aimed to investigate the changes in taste attributes, textural properties, bioactive compound contents, and aroma characteristics following thermal processing, and to clarify the underlying differences induced by distinct cooking techniques. Through comprehensive comparative analysis, this study seeks to deepen the understanding of chemical and sensory transformations occurring during pumpkin cooking and to provide a scientific basis for optimized processing strategies and consumer-oriented product development.

## 2 Materials and methods

### 2.1 Plant materials and thermal treatments

**Fig. 1:**
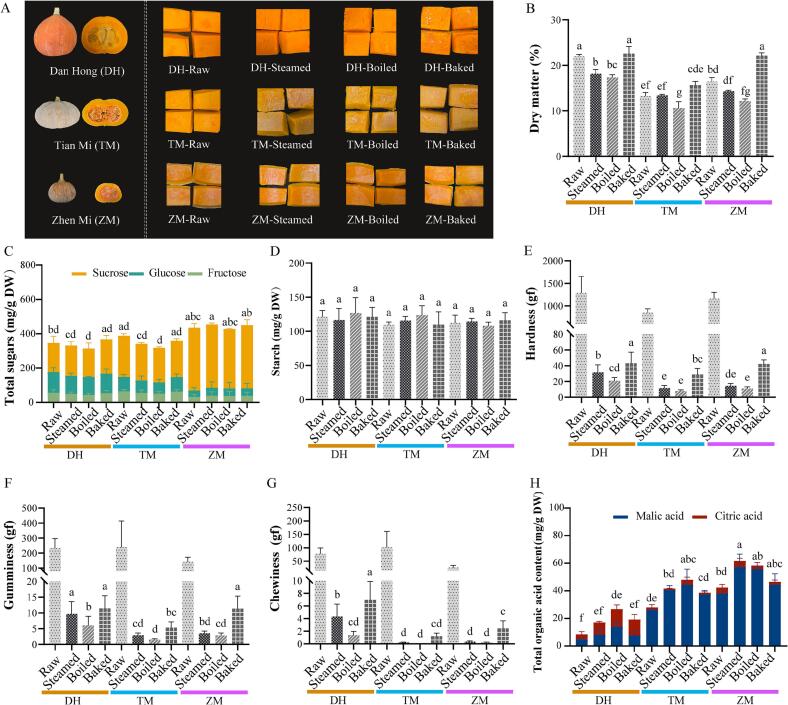
Effects of different cooking methods (boiling, steaming, and baking) on the taste and mouthfeel attributes of the *Cucurbita moschata* cultivars Zhen Mi (ZM) and Tian Mi (TM) and the *C. maxima* cultivar Dan Hong (DH). (A) Phenotypic appearance of raw and cooked samples; (B) Dry matter content; (C) Total sugars (including sucrose, fructose, and glucose); (D) Starch content; (E–G) Texture parameters: hardness, chewiness, and gumminess; (H) Organic acids (including malic acid and citric acid). Data are presented as the means ± standard deviations (*n* = 3). Different letters indicate significant differences among different cooking treatments for each cultivar (Tukey's test, *p* < 0.05).

This study utilized three pumpkin cultivars: Dan Hong (DH), a typical cultivar of *Cucurbita maxima*; Tian Mi (TM), an improved Miben (sweet-flesh) type of *Cucurbita moschata*; and Zhen Mi (ZM), a *C. moschata* cultivar with smaller fruit size and genetic background partially derived from *C. pepo* (Fig. 1A). They were harvested at 50 days after pollination from the experimental fields of the Vegetable Research Institute at the Guangdong Academy of Agricultural Sciences in Guangzhou, China (23°N, 113°E). During the cultivation of pumpkin, standard agrotechnical and care procedures, including irrigation and weeding, were performed. The primary sample consisted of 3 fruits from 3 individual plants of each cultivar. Pumpkin fruits were washed, deseeded, and cut into cubes with an average size of 35 × 25 mm. The prepared fruit cubes were subjected to three different thermal treatments: boiling, steaming, and baking. Boiling and steaming were conducted at 100 °C for 13 min, and baking at 200 °C for 20 min, using conditions similar to those reported by Piepiórka-Stepuk et al. (2023). These conditions were within the typical range for achieving edible pumpkin, and preliminary trials ensured that each treatment avoided undercooking or excessive tissue degradation, while reflecting common household cooking practices.

### 2.2 Determination of dry matter

All raw and cooked samples were immediately collected in liquid nitrogen and stored at −80 °C. The fruit pulp was then dried using a vacuum freeze-dryer (LaboGene, Denmark), finely ground into a powder, and preserved at −80 °C for subsequent physicochemical analysis. The dry matter content was calculated as the ratio of the sample's dry weight after freeze-drying to its fresh weight before freeze-drying, expressed as a percentage. Three biological replicates were conducted.

### 2.3 Determination of starch content

The freeze-dried samples were ground into a fine powder for starch extraction using iodine colorimetric method with slight modifications (Vainio, 1968). Specifically, 20 mg of freeze-dried pumpkin powder was extracted with 0.8 g/mL calcium nitrate solution. After extraction, 0.01 N iodine–potassium iodide (I₂-KI) reagent was added, and the absorbance was measured at 620 nm using a microplate spectrophotometer. Three biological replicates were conducted.

### 2.4 Determination of soluble sugar

Soluble sugars were determined following a previously described protocol (Xue et al., 2022). Briefly, the sugar profile of the lyophilized pumpkin powder samples was analyzed using high performance liquid chromatography (Alliance e2695 HPLC system, Waters, Milford, MA, United States) coupled with a refractive index detector and a medium-polarity NH2 column (Waters Xbridge BEH Amide-4.6 × 250 mm, 5 μm particle size). The samples were extracted with 50% acetonitrile water, and the mobile phase consisted of acetonitrile/deionized water (80:20 *v*/v) at a flow rate of 1 mL/min. Calibration standards for glucose, fructose, and sucrose were purchased from Sigma-Aldrich (St. Louis, MO, United States). Three biological replicates were conducted.

### 2.5 Determination of organic acids

Organic acids, including malic acid and citric acid, were detected following a previously described protocol (Xue et al., 2022). Briefly, High performance liquid chromatography (Alliance e2695 HPLC system, Waters, Milford, MA, United States) coupled with Waters 2998 photodiode array detector and reverse-phase C18 column (Waters Atlantis T3 C18 column, 250 mm × 4.6 mm i.d., 5 μm particle size) was applied to determine organic acids. The lyophilized pumpkin powder samples were extracted with distilled deionized water, and the mobile phase was a 97:3 *v*/v combination of 5 g/L (NH4)_2_HPO4 solution (pH 2.5) and methanol, with a flow rate of 0.6 mL/min. Eluted compounds were detected by UV absorbance at 214 nm. Calibration standards for malic acid and citric acid were obtained from Sigma-Aldrich (St. Louis, MO, United States). Three biological replicates were conducted.

### 2.6 Texture profile

The hardness, as a texture attribute, of all treatments (raw, steamed, boiled, and baked) of pumpkin was measured using a texture analyzer (TA-XTC-18, BosinTech Co., Ltd., Shanghai, China) equipped with a TA/2 probe. The analyzer settings were as follows: trigger force of 5 gf, with pre-test, test, and post-test speeds set to 4, 1, and 4 mm/s, respectively (Long et al., 2024). Three biological replicates were conducted.

### 2.7 Determination of carotenoids

The carotenoids were extracted and determined as described previously (Zhong et al., 2011). High performance liquid chromatography (Alliance e2695 HPLC system, Waters, Milford, MA, United States) coupled with Waters 2998 photodiode array detector and C18 column (Waters Spherisorb® ODS2 (4. 6 × 250 mm, 5 μm) was applied to determine carotenoids contents. The lyophilized pumpkin powder samples were extracted with acetone, and gradient elution was performed from 100% solvent A (acetonitrile/methanol/0.1 M Tris-HCl, pH 8.0, 84:2:14, *v*/*v*/v) to 100% solvent B (methanol/ethyl acetate, 68:32, v/v) over 15 min, followed by 10 min of 100% solvent B, and a 3-min re-equilibration with 100% solvent A, with a flow rate of 1.2 mL/min. Carotenoids were estimated spectrophotometrically (at 450 nm). Lutein, violaxanthin, zeaxanthin, α-carotene, and β-carotene were purchased from Sigma (St Louis, MO, USA). Three biological replicates were conducted.

### 2.8 Determination of vitamin C

The vitamin C was extracted and determined as described previously (Sajjad et al., 2024). Vitamin C was extracted using a solution containing 3% metaphosphoric acid, 8% glacial acetic acid, and 1 mM ethylenediaminetetraacetic acid. The extracts were analyzed by HPLC (Alliance e2695, Waters, USA) equipped with a photodiode array detector (Waters 2998) and a Waters Atlantis T3 C18 column (4.6 × 250 mm, 5 μm). Separation was carried out with 0.1% formic acid as the mobile phase at a flow rate of 1 mL/min, column temperature of 25 °C, and injection volume of 20 μL. Detection was performed at 245 nm, and quantification was based on an l-ascorbic acid standard curve (Sigma-Aldrich, Germany). Three biological replicates were conducted.

### 2.9 Antioxidant activity

Antioxidant capacity was determined using DPPH assay (1,1-diphenyl-2-picrylhydrazyl free radical) following the procedure of DPPH free radical scavenging assay kit (RS0128W) provided by Hongshuolin Biotechnology Co., Ltd., Guangzhou, China. The absorbance of the solution was measured at 517 nm, with methanol used as the solvent. A standard curve was generated using various concentrations of Trolox as the standard. Three biological replicates were conducted.

### 2.10 Measurement of volatile flavor compounds

Volatile analysis was conducted, following the previously reported protocol with some modifications (Liu et al., 2025). Briefly, 0.5 g of lyophilized powder and 2.5 ml of saturated sodium chloride solution were accurately placed into a 20 mL glass vial for solid phase microextraction (SPME). Subsequently, internal standard of 1 μL 3-nonanone (0.08 μg/μL, chromatographic ethanol as solvent) was added into the vial. The glass vial was placed in 70 °C agitator for 5 min. Afterward, an Agilent DVB/C-WR/PDMS/10 SPME fiber was inserted into the headspace of the sample for 30 min extraction. Before use, the SPME fiber was cleaned at 250 °C for 5 min. The SPME fiber was quickly inserted into the GC injection port after extraction for 5 min of desorption. The chromatographic analysis was performed using an Agilent 8860-5977C GC–MS with a split-splitless injector. The operation was performed in splitless mode. The capillary column was DB-5MS (60 m length, 0.25 mm I.D., 1 μm film thickness). The injector temperature was 270 °C. Carrier gas (He, 99.999%) flow rate: 1.8 mL/min. The temperature program was as follows: held at 50 °C for 5 min, ramped to 150 °C at 5 °C/min and held for 2 min, then ramped to 214 °C at 5 °C/min and held for 3 min. Finally, it was ramped to 250 °C at 5 °C/min and held for 6 min. The temperature of the electron ionization source: 230 °C. Ionization voltage: 70 eV. Data acquisition range: 40–350 *m*/*z*. Volatile compounds were identified by comparing mass spectra with the NIST 20 library and by matching calculated linear retention indices (RIs) with published reference values, using a homologous n-alkane series (C6–C21, Sigma-Aldrich, Shanghai, China). Quantification was performed using the internal standard method, and results were expressed as relative concentrations based on peak area normalization with 3-nonanone as the reference compound. Three biological replicates were conducted.

### 2.11 Statistical analysis

Statistical analyses were performed using GraphPad Prism 10. Two-way analysis of variance (ANOVA) was performed, followed by Tukey's Honestly Significant Difference (HSD) test for multiple comparisons. Differences were considered statistically significant at *p* < 0.05.

## 3 Result and discussion

### 3.1 Taste and mouthfeel

Dry matter, starch, sugars, and organic acids act as key contributors to the taste and mouthfeel of pumpkin. In this study, the dry matter content of raw DH (22%) was found to be 67% and 34% higher than that of raw TM and ZM, respectively. The different cooking methods had varying effects on the dry matter content of pumpkin: boiling tended to decrease the dry matter content, whereas baking tended to increase it. This pattern was consistent with previous findings from studies on pumpkin and sweet potato (Monalisa et al., 2019; Zhang et al., 2023) (Fig. 1B, Table S1). These changes were likely due to water absorption during boiling and moisture loss during baking.

Fructose, glucose, and sucrose are the major soluble sugars in pumpkin and are critical determinants of its taste. In this study, comparative analysis of the three cultivars showed that total sugar contents were 435.58, 346.26, and 388.14 mg/g DW in ZM, DH, and TM, respectively. ZM exhibited the highest sugar content, indicating a potential advantage with respect to sweet compound accumulation. Notably, the sucrose content of ZM (366.46 mg/g DW) was significantly higher than that of DH (169.63 mg/g DW) and TM (239.84 mg/g DW), representing a difference of approximately 116.0% and 52.8%, respectively (Fig. 1C, Table S1). Further analysis revealed that steaming, boiling, and baking had no significant impact on the composition or content of sugars across the three cultivars of pumpkin. Likewise, the starch content remained largely unchanged even after these cooking treatments (Fig. 1D, Table S1). Interestingly, although the total contents of sugars and starch remained similar, the perceived sweetness of the pumpkin cultivars appeared to increase after various cooking treatments. This observation was based on preliminary informal sensory assessments and is consistent with previously reported phenomena in fruits and vegetables (Allan et al., 2024), where structural and textural changes can modulate taste perception. The enhanced perception of sweetness may be attributed to the disruption of pumpkin cell walls during steaming, boiling, and baking, which facilitates the release and accessibility of soluble sugars, as well as to the softened texture, which may improve sugar diffusion and interaction with taste receptors.

Thermal treatments can profoundly affect the texture of fruits and vegetables, strongly influencing their sensory appeal and palatability. Texture encompasses characteristics such as smoothness, crunchiness, firmness, and creaminess, and, similar to flavor, is a key indicator of food quality and consumer satisfaction (Debnath et al., 2020). Cooking-induced softening results from several mechanisms, including starch gelatinization, hydrolysis, cell separation, loss of turgor, membrane disruption, and protein denaturation (Aboubakar et al., 2009). During steaming, boiling, and baking, pumpkin starch absorbs water, swells, and loses its ordered structure, forming a soft gel-like matrix. This irreversible heat-induced transformation strongly influences the texture of cooked starchy foods (Ai & Jane, 2015) and contributes to the softer texture and smoother mouthfeel of cooked pumpkin. Previous studies have confirmed that thermal processing significantly reduces the hardness, cohesiveness, resilience, and chewiness of pumpkin (Moreno et al., 2023; Rinaldi et al., 2021). In the present study, due to the relatively low moisture levels during baking, the extent of starch gelatinization appeared to be lower than that observed after steaming or boiling, often creating a firmer texture that was characterized by increased hardness, chewiness, and gumminess. Among the three cultivars, DH exhibited significantly higher hardness, chewiness, and gumminess after cooking than TM and ZM (Fig. 1E-1G, Table S1). These textural characteristics may influence consumer preference, providing a basis for cultivar selection for food processing or culinary applications.

Malic acid and citric acid, the main organic acids in pumpkin, play important roles in freshness and flavor balance. Nevertheless, excessive accumulation of these acids has been linked to reduced sensory quality, with taste scores showing a negative correlation with malic acid content in pumpkin (Xu et al., 2024). In this study, significant differences in organic acid content were observed among the three cultivars. Notably, the *C. moschata* cultivars ZM and TM contained markedly higher levels, 42.45 mg/g DW and 27.32 mg/g DW, respectively, compared with the *C. maxima* cultivar DH, which contained only 8.42 mg/g DW. Specifically, the organic acid content of ZM and TM was approximately 5.0 and 3.2 times that of DH, respectively. This suggested that these cultivars tended to accumulate more organic acids, potentially intensifying sourness and negatively affecting flavor perception. Moreover, all cooking methods increased the organic acid content, with steaming and boiling showing the most pronounced effects. Upon steaming, organic acids increased by approximately 45.5% (ZM), 52.2% (TM), and 103.2% (DH); after boiling, the respective increases were about 37.4%, 75.5%, and 217.4% (Fig. 1H, Table S1). Similar trends have been observed in other thermally processed vegetables, such as chestnuts, where boiling increases citric acid content (Gonçalves et al., 2010). Although one study reports increased pH after boiling (Monalisa et al., 2019), which contrasts with our results, the increases in organic acids can be explained by thermal disruption of the plant cell matrix and tissue softening, enhancing extractability, as well as heat-induced inactivation of enzymes, which prevents metabolic degradation and results in higher measured levels of malic and citric acids. Taken together, the observed increase in organic acids during cooking indicates that cultivars with higher initial acid levels may exhibit more pronounced sourness after thermal processing, potentially affecting sensory acceptability. This emphasizes that organic acid content should be considered a critical quality trait in cultivar selection, breeding program design, and the optimization of cooking methods to improve sensory quality.

### 3.2 Bioactive compounds and antioxidant activity

Pumpkin can serve as a nutritious complement to rice- and wheat-based staples due to its high carotenoid content. The vibrant color of its flesh is primarily derived from the varying proportions of violaxanthin, lutein, zeaxanthin, α-carotene, and β-carotene (Luo, Wang, Wang, et al., 2021). These abundant carotenoids not only help protect the plant from photodamage and photooxidative stress but also serve as major dietary sources of provitamin A for humans. Notably, these compounds play important roles in preventing retinal damage, cataract, and age-related macular degeneration, while also exhibiting antioxidant, anti-cancer, and anti-aging properties (Nisar et al., 2015).

**Fig. 2:**
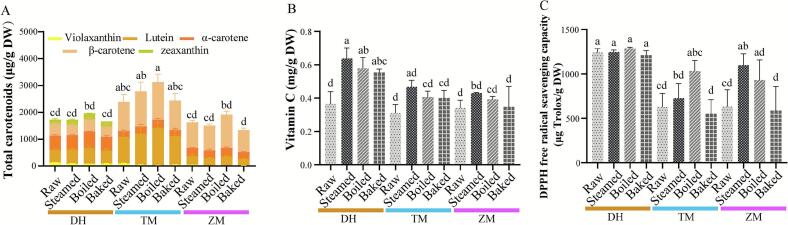
Effects of different cooking methods (boiling, steaming, and baking) on the bioactive compound content and antioxidant activity of the *Cucurbita moschata* cultivars Zhen Mi (ZM) and Tian Mi (TM) and the *C. maxima* cultivar Dan Hong (DH). (A) Total carotenoids (including violaxanthin, lutein, zeaxanthin, α-carotene, and β-carotene); (B) Vitamin C; (C) DPPH free radical scavenging capacity. Data are presented as the means ± standard deviations (*n* = 3). Different letters indicate significant differences among cooking treatments for each cultivar (Tukey's test, *p* < 0.05).

The results of this study showed that the lutein content of TM (986.23 μg/g DW) was 106.4% and 208.9% higher than that of DH (477.73 μg/g DW) and ZM (319.32 μg/g DW), respectively. Furthermore, thermal processing had a notable effect on lutein retention, especially in TM. Specifically, boiling enhanced the measured lutein levels of TM to 1401.02 μg/g DW, representing an approximate 42.0% increase when compared to raw TM. The α-carotene content of the *C. maxima* cultivar DH (521.43 μg/g DW) was significantly higher than that of the *C. moschata* cultivars TM (222.71 μg/g DW) and ZM (303.46 μg/g DW). Conversely, the β-carotene content of the *C. moschata* cultivars TM (1081.89 μg/g DW) and ZM (956.20 μg/g DW) was notably higher than that of the *C. maxima* cultivar DH (452.67 μg/g DW). In addition, zeaxanthin was exclusively detected in the *C. maxima* cultivar DH. Cooking enhanced the concentration of this compound, with boiling providing the greatest increase. However, cooking treatments had a minimal impact on the concentrations of α-carotene and β-carotene across the three cultivars. DH contained significantly higher levels of violaxanthin than TM and ZM. Cooking treatments, especially steaming and boiling, caused a marked decline in violaxanthin levels in the three cultivars, causing almost complete violaxanthin degradation in ZM (Fig. 2A, Table S1). This revealed that among the four major carotenoids found in pumpkin, violaxanthin was the most unstable. In summary, the TM cultivar demonstrated the highest total carotenoid accumulation among the tested varieties. Among the three cooking methods evaluated, boiling was the most effective at retaining and enhancing the content of lutein and total carotenoids in the pumpkin cultivars. This was likely because, despite causing possible isomerization and oxidation (Colle et al., 2016), thermal processing broke down the food matrix and loosened carotene-binding fibers, improving carotenoid extractability. Boiling resulted in the greatest tissue disruption, inducing cell wall rupture and turgor loss and thereby facilitating the most efficient release of carotenoids (Rinaldi et al., 2021). These findings are consistent with previous reports, which indicate that cooking can enhance the bioaccessibility and content of certain nutrients, including carotenoids (Moreira et al., 2020; Rinaldi et al., 2021). In line with other studies in potatoes, we found that lutein was the most stable carotenoid, whereas violaxanthin was the most vulnerable to degradation (Kotíková et al., 2016). These findings provide a scientific basis for recommending optimal cooking methods to preserve the nutritional quality of pumpkin.

Vitamin C, a key nutrient and antioxidant, showed no significant difference in content between *C. moschata* and *C. maxima*. In the raw state, the *C. maxima* cultivar DH had a vitamin C content of 0.37 mg/g DW. Following cooking, the content of vitamin C increased to varying extents: 0.64 mg/g DW after steaming, 0.58 mg/g DW after boiling, and 0.55 mg/g DW after baking, representing increases of 73.0%, 56.8%, and 48.6%, respectively. While two *C. moschata* cultivars also exhibited an increasing trend in vitamin C content after cooking, the differences were not statistically significant (Fig. 2B, Table S1). Steaming caused the most pronounced increase, which may result from heat-induced cell wall disruption and matrix softening, facilitating the release and extractable fraction of vitamin C (Palermo et al., 2014).

Finally, antioxidant activity assays revealed that the *C. maxima* cultivar DH had the highest DPPH free radical scavenging capacity of 1240.48 μg Trolox/g DW, which remained largely unchanged after cooking. In contrast, the *C. moschata* cultivars TM and ZM had initial DPPH free radical scavenging capacity values of 627.20 and 630.31 μg Trolox/g DW, respectively. Nevertheless, their antioxidant activity increased significantly after steaming or boiling, particularly in boiled TM (1031.90 μg Trolox/g DW; 64.52% increase) and steamed ZM (1099.34 μg Trolox/g DW; 74.40% increase), although baking had no such effect (Fig. 2C, Table S1). Previous studies on the DPPH radical scavenging activity of raw samples from different pumpkin species have shown that *C. maxima* exhibits significantly higher antioxidant capacity than *C. moschata*, consistent with the results of the present study (Zhou et al., 2017). As observed in this study, steaming and boiling can enhance the carotenoid content of pumpkin, partly explaining the increased antioxidant activity observed after these treatments, given that carotenoids are potent antioxidants. In the TM cultivar, a strong positive correlation was found between carotenoid content and DPPH radical scavenging capacity (*r* = 0.95). Moreover, the increase in antioxidant capacity may also be attributed to the heat-induced inactivation of pro-oxidant enzymes such as peroxidases (Jimenez-Monreal et al., 2009). Previous studies on pumpkin and other vegetable crops, have reported similar trends, consistently demonstrating that boiling enhances antioxidant activity (Buratti et al., 2020; Moreira et al., 2020; Rinaldi et al., 2021).

### 3.3 Volatile constituents

**Fig. 3:**
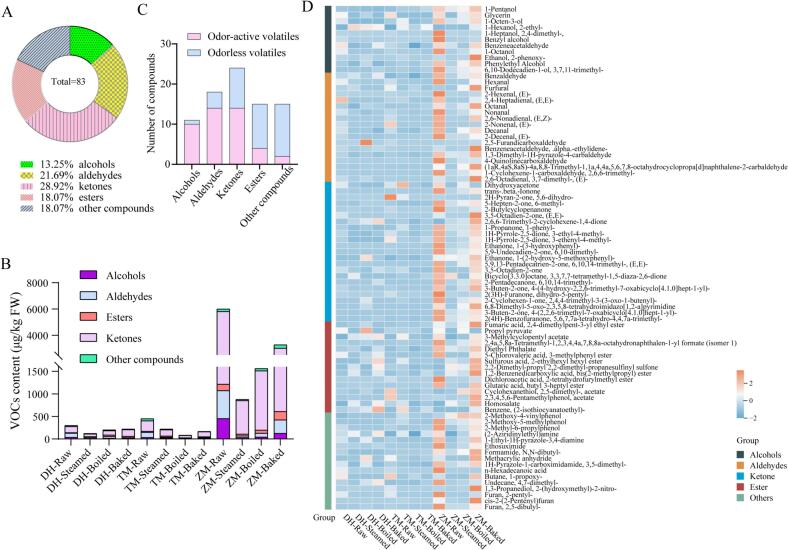
Volatile profiles of the *Cucurbita moschata* cultivars Zhen Mi (ZM) and Tian Mi (TM) and the *C. maxima* cultivar Dan Hong (DH) analyzed using headspace solid-phase microextraction coupled with gas chromatography–mass spectrometry (HS-SPME-GC–MS) to assess the effects of boiling, steaming, and baking. (A) Classification and quantification of volatile compounds. (B) Changes in volatile organic compounds in different cultivars after cooking. (C) Quantitative comparison of odor-active and odorless volatile compounds. (D) Heatmap visualization of all volatile compounds detected across different treatments.

Volatile organic compounds (VOCs) provide the characteristic aroma of foods and significantly enhance their palatability and consumer appeal. Alongside sugars, organic acids, salts, and other taste-active substances, VOCs play a crucial role in shaping the overall flavor profile of food products. In this study, a total of 83 volatile compounds were identified from pumpkin using headspace solid-phase microextraction-gas chromatography–mass spectrometry (HS-SPME-GC–MS), including alcohols (11 compounds, 13.25%), aldehydes (18, 21.69%), ketones (24, 28.92%), esters (15, 18.07%), and other compounds (15, 18.07%; e.g., phenols and furans) (Fig. 3A, Table S2). Among these, ketones were the most abundant VOCs. Comparative analysis showed that *C. moschata* contained higher levels of VOCs than *C. maxima*, with ZM exhibiting a significantly greater total volatile content than the other cultivars (Fig. 3B). This difference may be partially related to differences in primary metabolites, such as carbohydrates (e.g., sucrose) and organic acids, which serve as precursors in various volatile biosynthesis pathways. Evidence shows that the aroma of vegetables generally originates from primary nutrients such as carbohydrates, particularly mono- and disaccharides, proteins and free amino acids, fats and triglycerides or their derivatives, as well as vitamins and minerals, which are produced through photosynthesis and associated metabolic processes in the plant (Dresow & Böhm, 2009). In this study, ketones consistently accounted for the largest proportion of VOCs in all three cultivars both before and after cooking, with ZM exhibiting significantly higher levels of ketones across all treatments than the other two cultivars (Fig. 3B). Additionally, in both DH and ZM, the boiled and baked samples showed higher levels of alcohols, ketones, and esters than the steamed samples (Fig. 3B). Previous studies have identified alcohols, aldehydes, ketones, and esters as the predominant VOCs in raw pumpkin, in line with our findings. However, research on how cooking affects the VOC composition of pumpkin remains limited (Li et al., 2022; Zhou et al., 2017).

In addition, a total of 45 odor-active volatile compounds were identified in the pumpkin samples, including 10 alcohols, 14 aldehydes, 14 ketones, 4 esters, 1 phenol, and 1 furan (Fig. 3C). These volatiles contributed to a wide range of odor types, such as fermented, earthy, floral, green, fruity, bready, caramellic, citrus, minty, fatty, and aldehydic odors.

After cooking, the abundance of many compounds associated with green or earthy odors decreased in pumpkin. These included alcohols such as 1-octen-3-ol and 1-octanol; aldehydes such as hexanal, (E)-2-hexenal, (E,Z)-2,6-nonadienal, and (E)-2-nonenal; and ketones such as (E,E)-3,5-octadien-2-one (Fig. 3D). Among the “earthy” and “green” odorants, compounds such as 1-octen-3-ol, hexanal, (E,Z)-2,6-nonadienal, and (E)-2-nonenal exhibited odor activity values (OAVs >1) in raw pumpkin, indicating a significant contribution to the characteristic earthy off-flavor. Thermal processing substantially reduced the concentrations of these volatiles, with steaming and boiling exerting the greatest suppressive effects, in several cases decreasing their levels to below sensory threshold values (Table S2). Consequently, the attenuation of these high-impact odorants likely underlies the reduced perception of undesirable earthy/green notes in cooked pumpkin. These findings are consistent with previous observations in other heat-treated vegetables, where key green/earthy volatiles were also diminished after thermal processing (Garcia-Parra et al., 2020; Zhang et al., 2023). However, prior studies seldom compared the differential effects of distinct cooking methods, whereas our results clearly demonstrate method-dependent reductions in these odorants, highlighting the critical influence of cooking technique on aroma modulation in pumpkin.

In contrast, the contents of several pleasant aroma compounds with floral, fruity, bready, caramellic, and citrus notes increased in pumpkin after cooking (Parliment, 1989) (Fig. 3D, Table S2). Among aldehyde compounds, furfural is produced via the Maillard reaction (Tsai et al., 2021) and contributes bready and sweet aromas (Gu et al., 2022). Its levels increased after cooking, showing 1.76-fold in DH-steamed, 9.76-fold in ZM-boiled, and 25.34-fold in ZM-baked samples compared with raw samples. Notably, in ZM, baking significantly enhanced furfural formation, resulting in odor activity value (OAV) greater than 1, indicating that furfural became an aroma-active compound under this treatment and consequently contributed to a stronger burnt and roasted aroma. A similar pronounced increase in furfural was also reported in baked sweetpotato (Jiang et al., 2023).

In addition, the phenolic compound 2-methoxy-4-vinylphenol (also known as 4-vinylguaiacol), which imparts sweet, spicy, and smoky notes reminiscent of clove and vanilla, exhibited a marked increase after three cooking methods across all pumpkin varieties. Specifically, its levels increased by 5.51-fold in TM-steamed, 3.24-fold in ZM-steamed, 4.56-fold in ZM-boiled, and 4.27-fold in ZM-baked samples relative to the raw pumpkins, with all values exceeding their odor thresholds (Fig. 3D, Table S2). Notably, the ZM samples displayed the highest OAV, indicating a particularly strong contribution of 4-vinylguaiacol to the cooked aroma profile. 4-Vinylguaiacol is a widely reported volatile in thermally processed foods and fermented beverages, generated primarily through the thermal decarboxylation of ferulic acid (Ou & Kwok, 2004). Beyond its sensory significance, this compound has also attracted considerable attention due to its potential anticancer bioactivity (Luo, Wang, Sawadogo, et al., 2021; Tahir et al., 2024), highlighting its relevance not only to flavor quality but also to the functional attributes of food-derived phenolic volatiles.

Among the ester volatiles, propyl pyruvate is reported in FEMA and JECFA databases as having sweet, caramel-like, and floral notes; however, no reliable odor threshold values (in water or air) have been published. In this study, its concentrations increased after boiling across all three cultivars but declined under steaming and baking. Homosalate, exhibiting a balsamic and mildly menthol-like odor and similarly lacking reported threshold data, increased in all steamed samples. Due to the absence of threshold values, OAV calculation was not performed for these compounds, and their sensory contributions remain hypothetical, requiring validation through sensory evaluation or GC olfactometry analysis.

A comparative analysis of earthy/green and sweet, caramel-like, and bready odorants with OAV > 1 indicated that boiling was the most effective cooking method for simultaneously suppressing undesirable volatiles and enhancing desirable aroma contributors. Boiling also led to higher retention or enhancement of carotenoids, vitamin C, and antioxidant activity, indicating it as the optimal cooking approach for balancing nutritional quality and aroma. Taste-related analysis revealed that steaming and boiling significantly increased organic acid levels; since high organic acid content can negatively affect taste, these results support the use of molecular breeding strategies to lower excessive organic acids while preserving favorable sensory and nutritional traits.

## 4 Conclusions

This study demonstrates that thermal processing substantially modifies the sensory, nutritional, and aroma characteristics of *Cucurbita moschata* and *C. maxima*. Boiling and steaming enhanced carotenoids, vitamin C, and antioxidant activity while softening texture through starch gelatinization, although both methods also increased organic acid contents, which may negatively affect taste. Baking induced comparatively minor changes in physicochemical and nutritional attributes. Volatile analysis showed that cooking, particularly boiling, markedly decreased green and earthy odorants and promoted the formation of sweet, caramel-like, and bready aroma compounds such as furfural, 4-vinylguaiacol and propyl pyruvate. Integrating these observations, boiling emerged as the most favorable cooking method, providing the best overall balance between nutritional quality and desirable aroma. Differences in organic acids, carotenoids, and key odor-active volatiles across cultivars and cooking methods provide useful quality indicators for guiding cultivar selection, consumer-oriented cooking guidance, and breeding strategies aimed at improving flavor and nutritional value. This study is limited by the absence of sensory panel validation and bioaccessibility tests, which restrict direct conclusions on consumer perception and nutritional efficacy. Future studies incorporating sensory evaluation and digestion models are needed to confirm the practical relevance of these findings.

## CRediT authorship contribution statement

**Shudan Xue:** Writing – review & editing, Writing – original draft, Methodology, Funding acquisition, Formal analysis, Data curation, Conceptualization. **Yang Yu:** Investigation, Data curation. **Yingchao Xu:** Validation, Methodology. **Wenlong Luo:** Validation, Investigation. **Yingyin Lin:** Methodology, Data curation. **Muhammad Sajjad:** Methodology, Data curation. **Qingmin Jin:** Supervision, Methodology, Data curation. **Dasen Xie:** Supervision. **Yujuan Zhong:** Writing – review & editing, Validation, Supervision, Project administration, Investigation, Funding acquisition, Data curation.

## Declaration of competing interest

The authors declare that they have no known competing financial interests or personal relationships that could have appeared to influence the work reported in this paper.

## Data Availability

Data will be made available on request.

## Appendix A Supplementary data

Supplementary material Image 1
